# Komplexe kutane Leishmaniose mit ossärer Beteiligung

**DOI:** 10.1007/s00105-024-05355-2

**Published:** 2024-06-25

**Authors:** G. A. Hübner, W. Prüßmann, N. Jossifov, A. Mischnik, P. Terheyden

**Affiliations:** 1 Klinik für Dermatologie, Allergologie und Venerologie (Hautklinik), UKSH Lübeck, Ratzeburger Allee 160, 23538 Lübeck, Deutschland; 2 Klinik für Infektiologie und Mikrobiologie, UKSH Lübeck, Lübeck, Deutschland; 3 Gesundheitsamt Hansestadt Lübeck, Lübeck, Deutschland

**Keywords:** Erregerdiagnostik, Immunsuppression, Polymerasekettenreaktion, *Leishmania infantum*, Extrakutane Manifestationen, Pathogen diagnostics, Immunosuppression, Polymerase chain reaction, Leishmania infantum, Skin manifestations

## Abstract

Wir berichten über einen immunsupprimierten Patienten mit einer komplexen kutanen Leishmaniose (Erreger: *Leishmania* [*L.*]* infantum*), der eine ossäre Beteiligung des Digitus V des linken Fußes aufwies und erfolgreich mit Miltefosin behandelt wurde. Die Erregerdiagnostik erfolgte mittels positiver Polymerasekettenreaktion (PCR) in Haut- und Knochengewebe. Bei Immunsupprimierten sollte immer nach extrakutanen Manifestationen der Leishmaniose gesucht werden. Eine ossäre Beteiligung bei kutaner Leishmaniose mit *L. infantum* ist bisher noch nicht beschrieben worden.

## Abstract (en)

We report an immunocompromised patient with a complex cutaneous leishmaniasis (CL) who suffered from singular bone involvement of the little left toe due to Leishmania (L.) infantum infection. The diagnosis was confirmed by positive polymerase chain reaction (PCR) testing in skin and bone tissue. The patient was successfully treated with miltefosine. In immunocompromised patients with CL, extracutaneous manifestations should always be ruled out. This is the first case report describing osseous involvement in CL due to Leishmania infantum.

## Anamnese

Ein 52-jähriger Patient stellte sich im Juli 2019 mit Knoten im Gesicht und am Hals sowie einer schmerzhaften Rötung des Digitus V des linken Fußes vor. Diese Läsionen bestanden seit ca. 4 Monaten. Seit ca. 10 Monaten bestand zusätzlich ein Ulkus prätibial rechts. Das Allgemeinbefinden war bis auf Schmerzen im linken Zeh unbeeinträchtigt. Aufgrund einer rheumatoiden Arthritis erhielt der Patient seit 3 Jahren 20 mg Methotrexat subkutan wöchentlich. Die Reiseanamnese ergab jährliche Mittelmeerkreuzfahrten mit Aufenthalten auf Mallorca.

## Befund

**Abb. 1:**
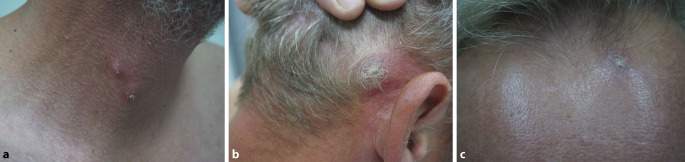
Hautbefund im Gesicht und am Hals: **a** Knoten am Hals, **b** Plaque retroaurikulär rechts, **c** Plaque an der Stirn

**Abb. 2:**
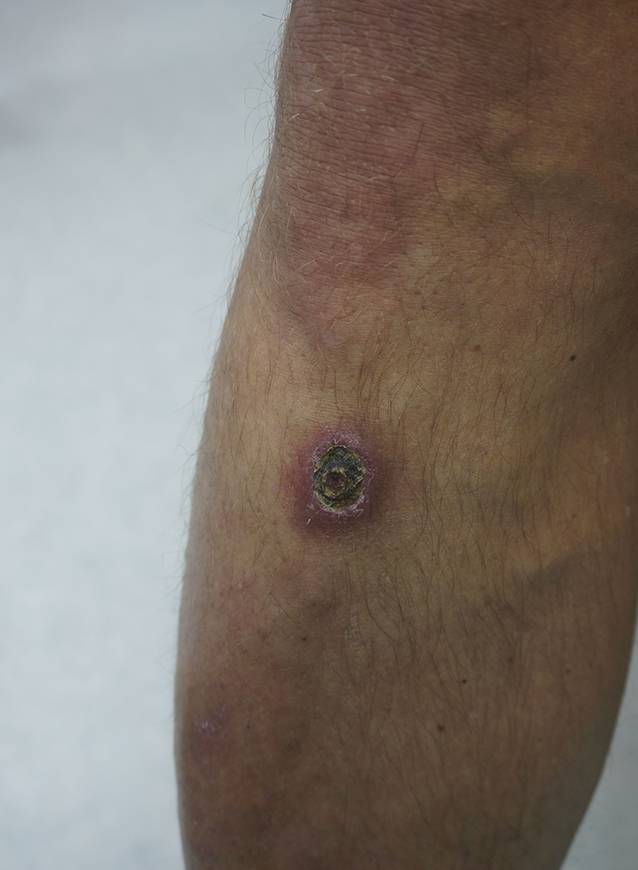
Ulzerierte Plaque prätibial rechts

**Abb. 3:**
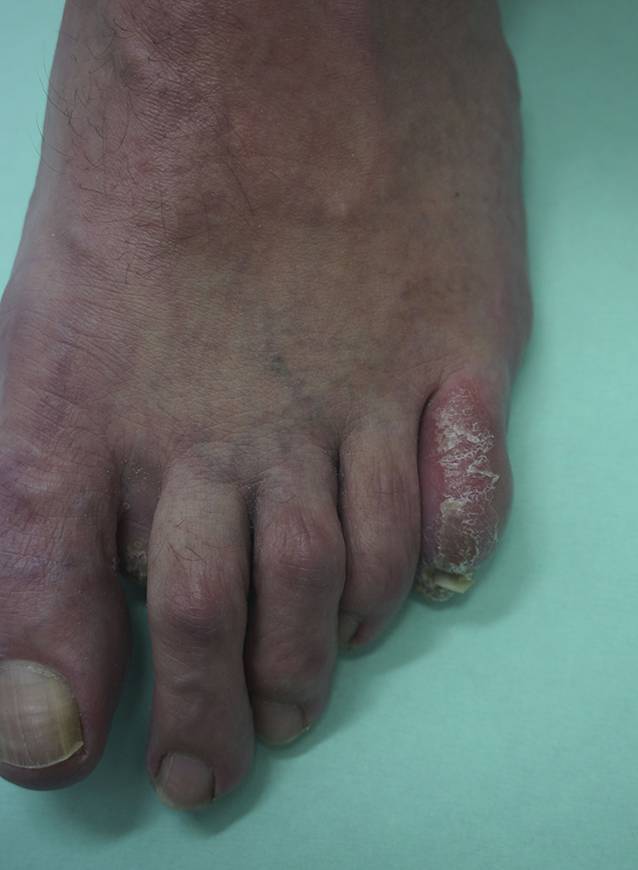
Kleinzehe des linken Fußes mit Ödem, Rötung und Schuppung

**Abb. 4:**
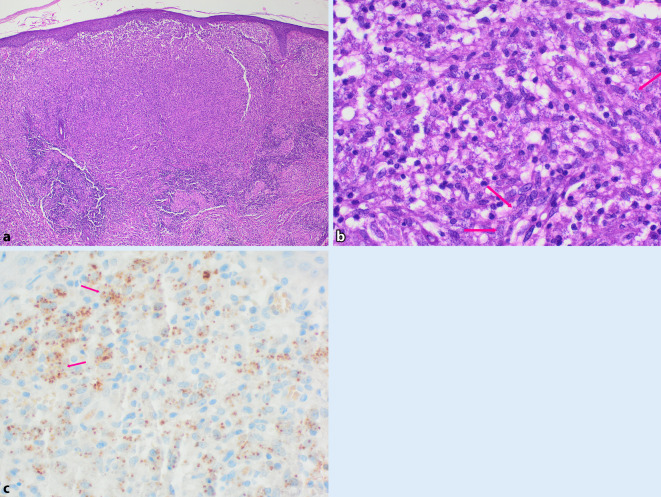
Histopathologie retroaurikulär rechts: **a** in der gesamten Dermis diffuses histiozytenreiches Entzündungsinfiltrat (HE [Hämatoxylin-Eosin], Vergr. 40:1), **b** Nachweis von Amastigoten in oberflächlichen Anteilen (HE, Vergr. 400:1), *Rote Pfeile*: Amastigoten, **c** Darstellung der Amastigoten mit der CD1a-Färbung (Vergr. 400:1). *Rote Pfeile*: Amastigoten

Am Hals rechts bestanden 2 erythematöse, schuppende Knoten (Abb. 1a). Submental, retroaurikulär rechts und an der Stirn zeigten sich Plaques mit verruköser Oberfläche (Abb. 1b, c). Prätibial rechts bestand eine zentral ulzerierte, schuppende Plaque (Abb. 2). Die linke Kleinzehe war geschwollen, gerötet und schuppte (Abb. 3). Histologisch zeigten sich in mehreren Biopsien vom Hals und retroaurikulär unter einem akanthotischen Epithel mit Parakeratose im gesamten Korium diffuse histiozytäre Entzündungsinfiltrate mit Beimischung von neutrophilen Granulozyten, Lymphozyten und Plasmazellen (Abb. 4a). In den oberflächlichen Anteilen konnten mit der Hämatoxylin-Eosin-Färbung (HE) intrazelluläre Amastigoten dargestellt werden (Abb. 4b), die sich immunhistochemisch positiv mit Anti-CD1a anfärben (Abb. 4c).

## Diagnose

**Abb. 5:**
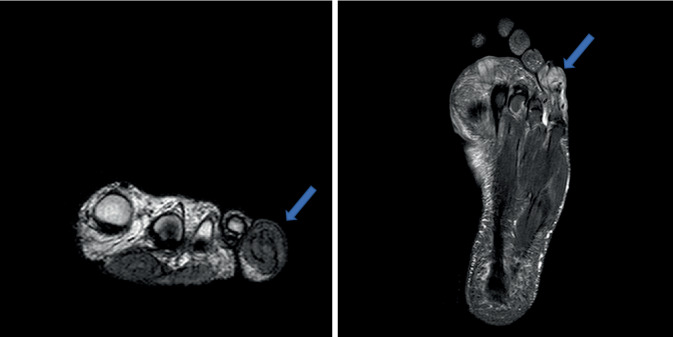
**a**, **b** Magnetresonanztomographie (STIR[Short-Tau-Inversion-Recovery]-Sequenz) des linken Fußes: Osteitis des Mittel- und Endgliedes Digitus V links (*Pfeile*), perifokale Weichteilschwellung, Ansicht von vorn (**a**) und plantar (**b**).

**Abb. 6:**
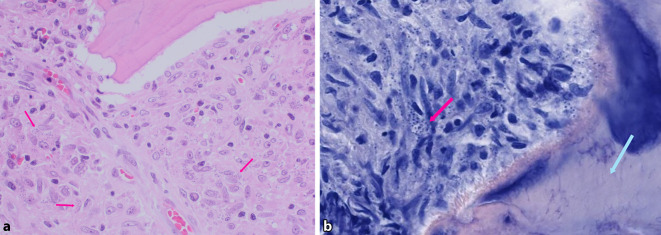
Histopathologie Mittelglied linke Kleinzehe: partiell granulomatöse Osteomyelitis mit Nachweis von Amastigoten (**a** Hämatoxylin-Eosin [HE], Vergr. 400:1, **b** Giemsa, Vergr. 400:1). *Rote Pfeile*: Amastigoten, *blauer Pfeil*: Trabekel

Der histologische Nachweis von Amastigoten sprach für eine kutane Leishmaniose. Aus den Läsionen im Gesicht konnte sowohl in den Schuppen als auch in einer Probebiopsie mittels Polymerasekettenreaktion (PCR) zum *Leishmania-donovani*-Komplex gehörende Nukleinsäure nachgewiesen werden; die Sequenzierung des parasitären *cpb*-Gens ergab *Leishmania* (*L.*) *infantum* (Bernhard-Nocht-Institut für Tropenmedizin, Hamburg). In den Hautschuppen der Kleinzehe konnten mittels PCR keine Leishmanien nachgewiesen werden. Die Magnetresonanztomographie (MRT) des linken Fußes zeigte ein Ödem mit begleitender Kontrastmittelaufnahme am Mittel- und Endglied D5; die angrenzenden Weichteile nahmen kräftig Kontrastmittel auf. Es wurde eine Osteitis des Mittel- und Endgliedes der Kleinzehe mit perifokaler Weichteilschwellung diagnostiziert (Abb. 5a, b). Bei Verdacht auf eine bakterielle Osteitis erhielt der Patient 3‑mal 600 mg Clindamycin oral. Aufgrund der ausbleibenden Besserung nach 6 Wochen Therapie wurde eine knöcherne Biopsie aus der linken Kleinzehe entnommen: Hier zeigte sich eine partiell granulomatöse Osteomyelitis (Trabekel mit Osteolysen, Granulationsgewebe, histiozytäre Proliferate) mit Nachweis von Amastigoten in den Entzündungszellen in der HE- und der Giemsa-Färbung (Abb. 6a, b). In der PCR aus dem Knochengewebe konnte ebenfalls *L. infantum* nachgewiesen werden. Somit konnte eine ossäre Beteiligung der kutanen Leishmaniose durch *L. infantum* diagnostiziert werden.

Die Thoraxröntgenaufnahme sowie eine Sonographie des Abdomens waren unauffällig. In der Computertomographie des Thorax zeigten leicht vergrößerte Lymphknoten mediastinal, hilär, paraaortal, parailiakal, retroperitoneal und inguinal. Laborchemisch fanden sich keine pathologischen Werte, die HIV(humanes Immundefizienzvirus)-Serologie war negativ.

## Therapie und Verlauf

**Abb. 7:**
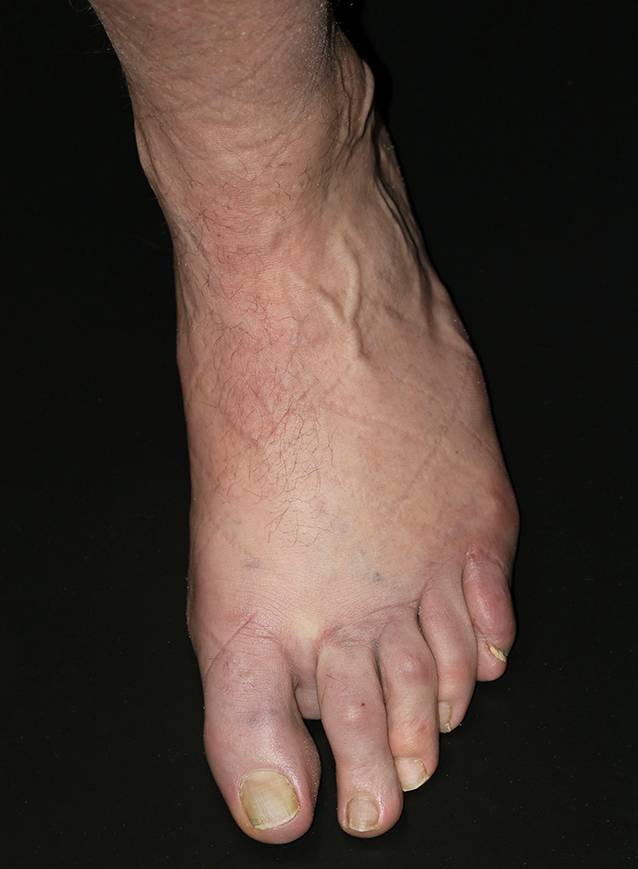
Abgeheilter Befund der linken Kleinzehe nach 6 Wochen Therapie mit Miltefosin

Initial therapierten wir intravenös mit liposomalem Amphotericin B (3 mg/kgKG [Körpergewicht]). Nach 4 Tagen entwickelte der Patient ein makulopapulöses Arzneiexanthem. Die Therapie wurde dann auf 3‑mal 50 mg Miltefosin täglich umgestellt und über 6 Wochen fortgesetzt. Unter der Therapie traten initial erhebliche Nebenwirkungen auf; es kam zu Müdigkeit, Nachtschweiß und Schüttelfrost. Zwei Monate nach Therapieende waren alle kutanen Herde abgeheilt. Die Kleinzehe links war nicht mehr gerötet oder geschwollen (Abb. 7), und es bestanden keine Schmerzen mehr. Ein Rezidiv zeigte sich bisher (3 ½ Jahre nach Therapieende) nicht.

## Diskussion

Die kutane Leishmaniose (CL) ist eine der am häufigsten nach Deutschland importierten Reisedermatosen [13, 16]. Der Erreger *Leishmania* gehört zu den Protozoen und zur Familie der Trypanosomen. Als Vektor fungiert die Sandmücke (Phlebotom). Das Reservoir sind v. a. Säugetiere (Hunde, Nagetiere) und Menschen [3, 5, 17].

Nach dem klinischen Bild wird zwischen CL (u. a. *L. infantum, L. tropica, L. major*), mukokutaner Leishmaniose (u. a*. L. braziliensis, L. guyanensis*) und viszeraler Leishmaniose (v. a. *L.-donovani*-Komplex) differenziert. Entsprechend der geografischen Verbreitung unterscheidet man zwischen der Leishmaniose der „Alten Welt“ (Europa, Afrika, Asien) und der Leishmaniose der „Neuen Welt“ (Mittel- und Südamerika) [3, 5, 13, 17].

Bei der „klassischen“ CL der „Alten Welt“ entwickelt sich aus einer Papel an der Einstichstelle ein Knoten, der dann häufig ulzeriert (sog. „Orient-Beule“). Die Abheilung erfolgt in den meisten Fällen nach einigen Monaten, spätestens nach 2 Jahren unter Narbenbildung [3, 5, 17].

Die Diagnose der CL stützt sich hauptsächlich auf die Histologie mit dem intrazellulären Nachweis von Amastigoten. Insbesondere bei länger bestehenden Läsionen sind diese aber oft nicht nachweisbar. In unserem Fall konnten sie in allen Läsionen, auch in den Anteilen der Osteomyelitis, mithilfe der HE- und der Giemsa-Färbung nachgewiesen werden. In den kutanen Läsionen wandten wir zusätzlich die immunhistochemische Anti-CD1a-Färbung an. Eine Anti-CD1a-Färbung (mittels MTB1-Klon) kann durch das Hervorheben der Amastigoten epidermal und subepidermal in der Hautbiopsie diagnostisch wegweisend sein [7, 9].

Finden sich in der Histologie epitheloidzellige Granulome, sollte neben anderen Erkrankungen (u. a. Tuberkulose, atypische Mykobakteriose, Sporotrichose, tiefe Trichophytie, Lepra, Sarkoidose) stets eine Leishmaniose in die Differenzialdiagnose einbezogen werden [3, 5, 11, 17]. Die PCR ist die sensitivste Methode des Erregernachweises und dient auch der Bestimmung der Spezies [3, 5, 17]. Aus dem Knochenmaterial der linken Kleinzehe konnte mittels PCR *L. infantum* nachgewiesen werden. Eine mögliche Kontamination bei der Entnahme der Probe durch *L.-infantum*-Antigen aus der darüber liegenden, läsionalen Haut ist denkbar. Die PCR der Schuppen des Kleinzehs war jedoch negativ. Beweisend für die durch die Leishmanien-Infektion verursachte Osteomyelitis ist die in der Histologie nachweisbare granulomatöse Osteomyelitis mit dem Nachweis von Amastigoten im Entzündungsinfiltrat durch die HE- und die Giemsa-Färbung.

Der identifizierte Erreger *L. infantum* gehört zum *L.-donovani*-Komplex und kann die viszerale, die CL sowie äußerst selten auch die mukokutane Leishmaniose verursachen [2].

Wahrscheinlich hat unser Patient die Infektion im Rahmen seiner jährlichen Kreuzfahrten im Mittelmeerraum erworben, aus dem *L. infantum* am häufigsten nach Deutschland importiert wird [8]. In den letzten 2 Jahrzehnten wurde zunehmend über Fälle von durch *L. infantum *ausgelöster CL in den Endemiegebieten berichtet [6, 11, 12, 15]. Bei CL mit Nachweis von *L. infantum* und gleichzeitig vorhandener Immundefizienz wird eine Knochenmarkbiopsie mit Erregerdiagnostik zum Ausschluss einer disseminierten Infektion empfohlen [3]. Diese lehnte der Patient ab. Der unauffällige Befund in der Sonographie des Abdomens, die unauffälligen laborchemischen Parameter (u. a. großes Blutbild, Leber‑, Nierenparameter und C‑reaktives Protein) sowie der gute Allgemeinzustand des Patienten sprachen klinisch gegen eine viszerale Leishmaniose.

Die ausgedehnten kutanen Läsionen und die ossäre Beteiligung waren sehr wahrscheinlich der Immunsuppression des Patienten geschuldet [1]. *Bei L.-infantum-*Infektionen ist bei Immunsuppression eher als bei anderen Erregern mit einer Disseminierung des Erregers zu rechnen [17]. Die bei unserem Patienten beschriebene Knochenbeteiligung bei kutaner Leishmaniose ist bisher nur einmal und ohne Speziesbestimmung beschrieben [10]. Es wurde histologisch eine granulomatöse Osteitis gesehen, der Nachweis von Leishmanien mithilfe der Giemsa-Färbung im ossären Entzündungsinfiltrat gelang. Bei einer viszeralen Leishmaniose ist ein indischer Fall mit einer Manifestation im Femur beschrieben [14].

Unser Fall erfüllte mehrere Kriterien einer komplexen CL: Es bestanden multiple Herde, die sich an sensiblen Bereichen wie Gesicht und Hals manifestierten. Die Therapie der komplexen CL sollte immer systemisch erfolgen [3–5]. Auch aufgrund des ossären Befalls musste eine systemische Therapie erfolgen. Miltefosin ist ein wirksames, oral verfügbares Präparat, das schon 2011 in der Leitlinie zur Therapie der komplexen kutanen Leishmaniose empfohlen und erfolgreich bei der kutanen Leishmaniose eingesetzt wurde [3, 4, 12]. Die Zulassung besteht aktuell jedoch ausschließlich für kutane Leishmaniosen der „Neuen Welt“ (also z. B. *L.-braziliensis*-Komplex) ab dem 12. Lebensjahr [4]. Bei der viszeralen Leishmaniose ist Miltefosin ab dem 3. Lebensjahr zugelassen.

Der Zusammenhang zwischen viszeraler Leishmaniose und CL bei Infektionen durch *L. infantum* sollte Gegenstand von Untersuchungen werden [6]. Insbesondere ist nicht geklärt, ob und wie oft Übergänge der kutanen in eine viszerale Form vorkommen [5, 6].

## Fazit für die Praxis

- Kutane Leishmaniosen aus dem Mittelmeerraum werden häufig durch *Leishmania* (*L.*)* infantum* verursacht.
- Eine Speziesdiagnostik mittels Polymerasekettenreaktion (PCR) ist anzustreben.
- Bei Immunsuppression sollte nach extrakutanen Manifestationen der kutanen Leishmaniose gefahndet werden. Die bei unserem Patienten diagnostizierte Knochenbeteiligung bei kutaner *L.-infantum*-Infektion wurde noch nicht beschrieben.
- Therapeutisch sind komplexe kutane Leishmaniosen immer systemisch zu behandeln.
